# Reaction of hydroxyl-quinoline with pentafluoropyridin

**DOI:** 10.1186/s40064-016-3410-z

**Published:** 2016-11-22

**Authors:** Khalil Beyki, Malek Taher Maghsoodlou, Reza Heydari

**Affiliations:** Department of Chemistry, Faculty of Science, University of Sistan and Baluchestan, P. O. Box 98135-674, Zahedan, Iran

**Keywords:** Pentafluoropyridine, Synthesis, Hydroxyl-quinoline, ^19^F-NMR

## Abstract

Reaction of pentafluoropyridine with 2 or 8-hydroxyl-quinoline under basic conditions in acetonitrile gives 4-oxy quinoline 2,3,5,6-tetrafluoropyridine derivatives in good yields. All the compounds were characterized using ^1^H, ^13^C, ^19^F-NMR and MS spectroscopy.

**Electronic supplementary material:**

The online version of this article (doi:10.1186/s40064-016-3410-z) contains supplementary material, which is available to authorized users.

## Background

The unique properties of fluorine atom make organofluorine compounds find many different applications, ranging from pharmaceuticals and agrochemicals to advanced materials and polymers. Circa 20 % of pharmaceuticals contain a fluorine atom (Hunter 2010; Champagne et al. 2015). The fluorinated groups in these systems lead to remarkable changes in their physical properties, chemical reactivity, and physiological activity (Iwao 2009). Pentafluoropyridine, as one of the simplest members of electron-deficient species of perfluoroheteroaromatic compounds, has been investigated into since the early 1960s (Fox et al. 2013). The most important reaction of pentafluoropyridines involves the replacement of the para-fluorine atom by nucleophilic reagents for the synthesis of new organofluorine compounds, such as heterocyclic and macrocyclic perfluoro systems (Cartwright et al. 2010; Chambers et al. 2005; Ranjbar-Karimi et al. 2015). In this paper, we have recently reported the reaction of pentafluoropyridine with hydroxyl-quinoline. This allows the synthesis of a wide range of 4-substituted 2,3,5,6-tetrafluoropyridine (Additional file 1).

## Results and discussion

**Fig. 1:**
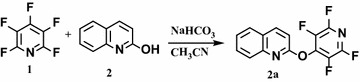
Reaction of pentafluoropyridine with 2-hydroxyl quinoline

In this short report, we describe nucleophilic substitution of pentafluoropyridine with 2 or 8-hydroxyl-quinoline and how the resulting products of 4-quinoline-perfluoropyridine derivatives. Reaction of pentafluoropyridine **1** with 2-hydroxyl quinoline **2** under basic conditions (NaHCO_3_) in acetonitrile at reflux temperature gave a single product of 2-(perfluoropyridin-4-yloxy)quinoline **2a** (Fig. 1). In 2-hydroxyl quinoline **2**, the hydroxyl group deprotonate by base and attacks at the most active para position of pentafluoropyridine to give **2a**.

The structures of **2a** were characterized by ^19^F, ^1^H, ^13^C NMR and mass spectra. In ^19^F NMR spectroscopy of **2a** observed two peaks for fluorine’s, a peak is observed as multiple at δ = −86.4 for fluorine atom located in the ortho position towards the ring nitrogen and also, a multiple is remarked at up field δ = −154.8 for fluorine atom located in the meta position towards the ring nitrogen.

The two resonances by ^19^F NMR and their chemical shift of them indicate that displacement of fluorine atoms attached to the para position of pyridine ring. In the ^1^H NMR spectrum of compound **2a**, the aromatic proton resonances were observed as doublets at δ = 7.01–8.01 ppm. Other spectroscopic techniques were consistent with the structures proposed. The mass spectrum of **2a** compound displayed molecular ion peaks at peak (M-1) at m/z = 293, and any initial fragmentation involved the loss of the other molecules which is consistent with the proposed structure.

**Fig. 2:**
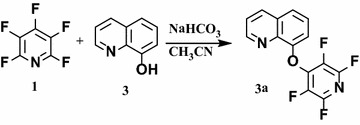
Reaction of pentafluoropyridine with 8-hydroxyl quinoline

Also, we examined the reaction of pentafluoropyridine **1** with 8-hydroxyl quinoline **3** in the presence of sodium hydrogen carbonate in CH_3_CN as a solvent gave 8-(perfluoropyridin-4-yloxy) quinoline **3a** (Fig. 2). In basic condition, hydroxyl group of the quinoline deprotonation and attack to *Para* position of pentafluoropyridine and elimination of 4-fluor pyridine ring to give **3a**. The purification of **3a** was achieved by column chromatography using ethyl acetate/n-hexane (1:8).

8-(perfluoropyridin-4-yloxy) quinoline **3a** was characterized by ^19^F NMR, in which the resonance attributed to the fluorine located in the ortho position towards the ring nitrogen has a chemical shift of −65.7 ppm and the fluorine resonance located in meta position occurs at −139.1 ppm. In ^1^H NMR of **3a**, the spectra protons of the aryl ring were observed at 6.8–7.9 ppm. The mass spectrum of **3a** displayed the molecular ion peak (M^+^) at m/z = 294, which is consistent with the proposed structure. Other spectroscopic techniques were consistent with the structures proposed.

## Conclusion

In conclusion, we showed that hydroxyl group in quinoline can react with pentafluoropyridine to afford of 2,3,5,6-tetrafluoropyridine quinoline characterized spectroscopically.

## Experimental

All materials and solvents were purchased from Merck and Aldrich and were used without any additional purification. Mass spectra were taken by a Micro mass Platform II: EI mode (70 eV). Silica plates (Merck) were used for TLC analysis.

### Typical procedure for preparation of 4-oxy quinoline 2,3,5,6-tetrafluoropyridine

Pentafluoropyridine (0.17 g, 1 mmol), hydroxyl-quinoline (0.16 g, 1 mmol) and sodium hydrogen carbonate (0.08 g, 1.0 mmol) were stirred together in CH_3_CN (5 mL) at reflux temperature for 4 h. After completion of the reaction (indicated by TLC), reaction mixture was evaporated to dryness and water (10 mL) was added and extracted with dichloromethane (2 × 10 mL) and ethylacetate (2 × 10 mL). The mixture was filtered, volatiles evaporated and the residue purified by column chromatography on silica gel using ethyl acetate/n-hexane (1:8).

**2-(perfluoropyridin-4-yloxy)quinoline 2a** (0.2 g, 77 %) as yellow solid; mp 195 °C; ^1^H NMR (DMSO): δ (ppm) 7.01–8.01 (6H, m, Ar–H). ^19^F NMR (CDCl_3_): δ (ppm) −86.4 (2F, m, F-2,6), −154.8 (2F, m, F-3,5).^13^C NMR (CDCl_3_): δ (ppm) 110.9, 112.6, 113.3, 122.3, 123.3, 128.7, 130.8, 148.2, 156.0, 161.1, 163.9, 165.1 MS (EI), m/z (%) = 293 (M^+^−1) 275, 253, 235, 213, 147, 83, 43.

**8-(perfluoropyridin-4-yloxy) quinoline 3a** (0.23 g, 80 %) as brown solid; mp 180 °C; ^1^H NMR (DMSO): δ (ppm) 6.84–7.92 (6H, m, Ar–H). ^19^F NMR (CDCl_3_): δ (ppm) −65.7 (2F, m, F−2,6), −139.1 (2F, m, F−3,5). ^13^C NMR (DMSO): δ (ppm) 110.9, 112.6, 113.3, 122.3, 123.3, 128.7, 130.8, 131.3, 148.2, 150.0, 161.1, 163.9, 165.1 MS (EI), m/z (%) = 294 (M^+^) 282, 275, 246, 227, 167, 122, 101, 85, 58, 43.

## Additional file

**Additional file 1.** ^1^H, ^13^C, ^19^F-NMR and MS spectra of the compounds.
