# Supplementary figures and images for: Reaction of hydroxyl-quinoline with pentafluoropyridin
Source: Springerplus. 2016 Nov 22;5(1):1996. doi: 10.1186/s40064-016-3410-z (PMC5118374; doi:10.1186/s40064-016-3410-z)

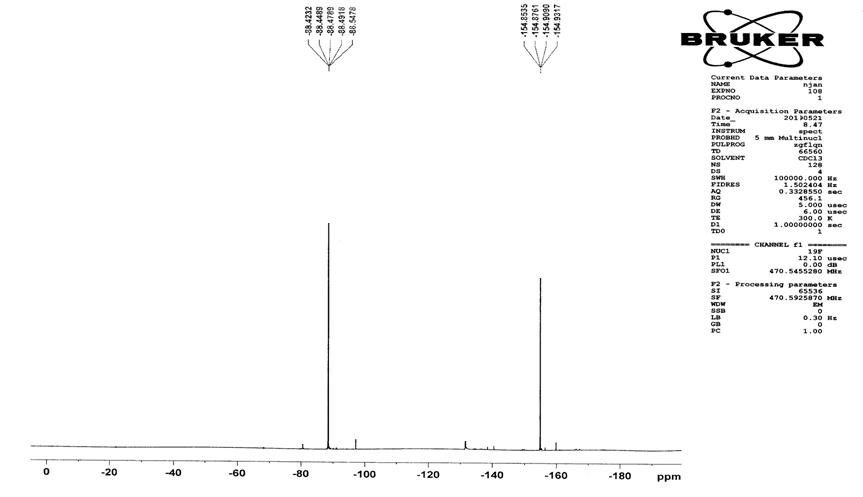


19F-NMR **2a**


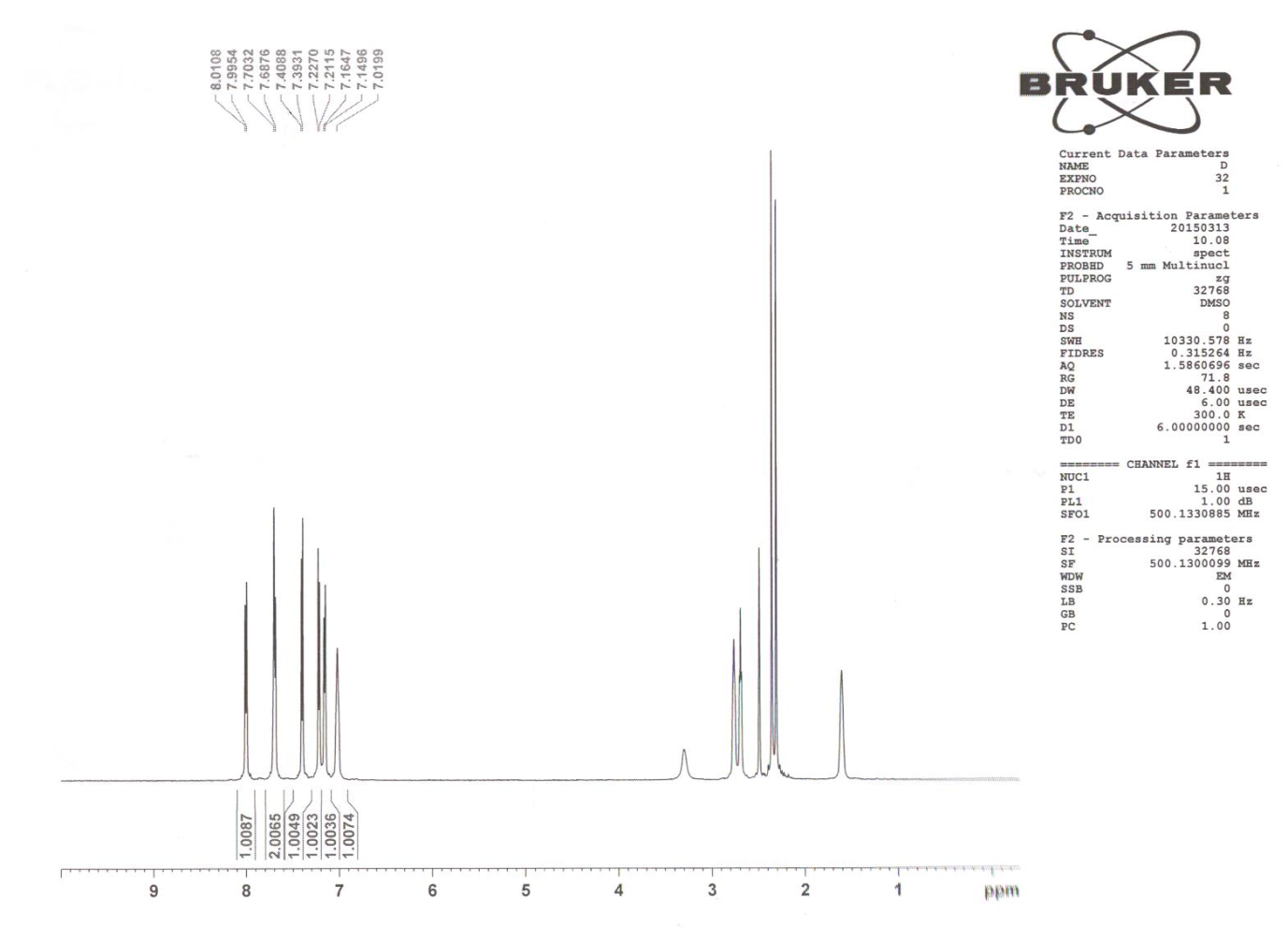


1H-NMR **2a**


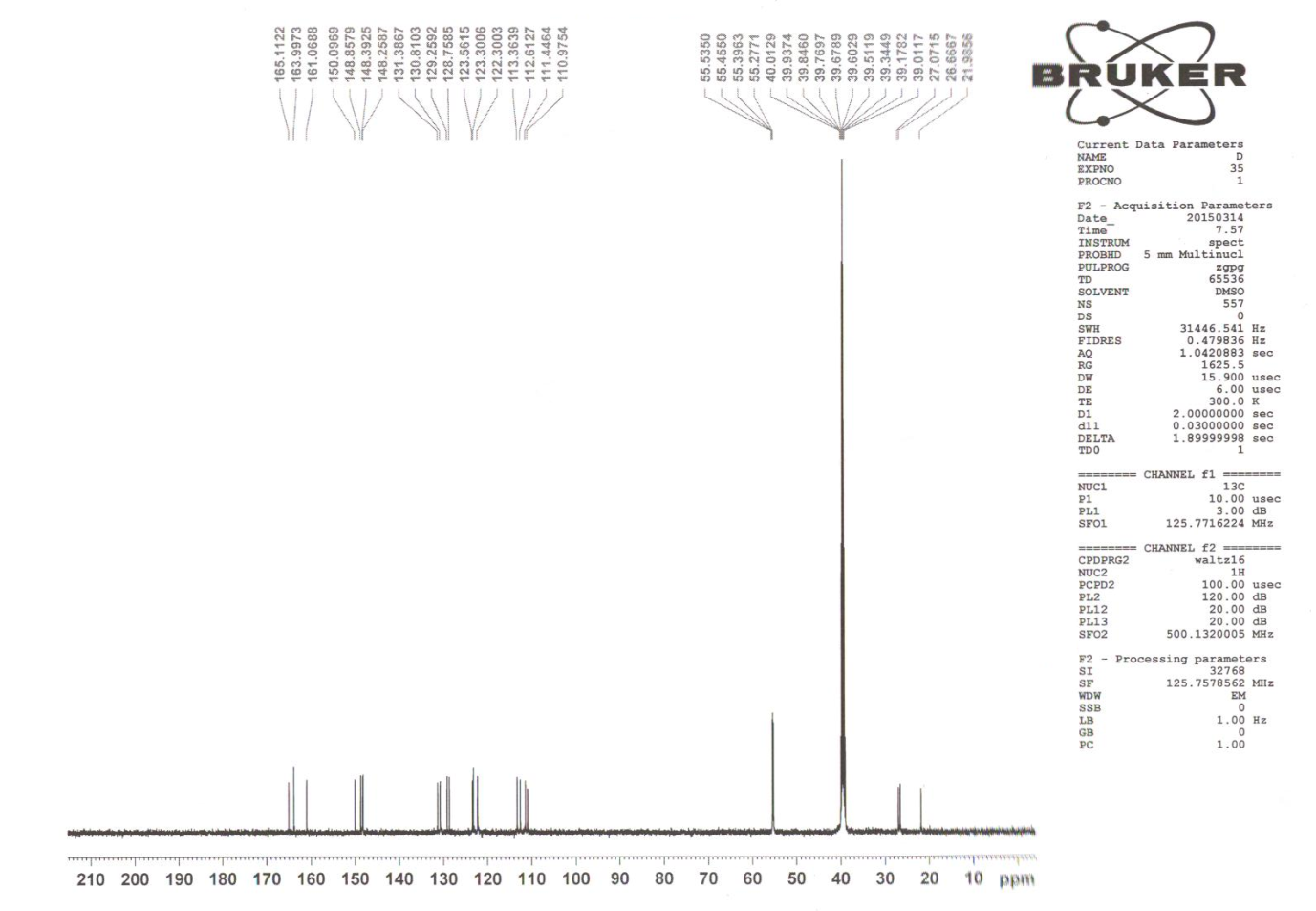


13C-NMR **2a**


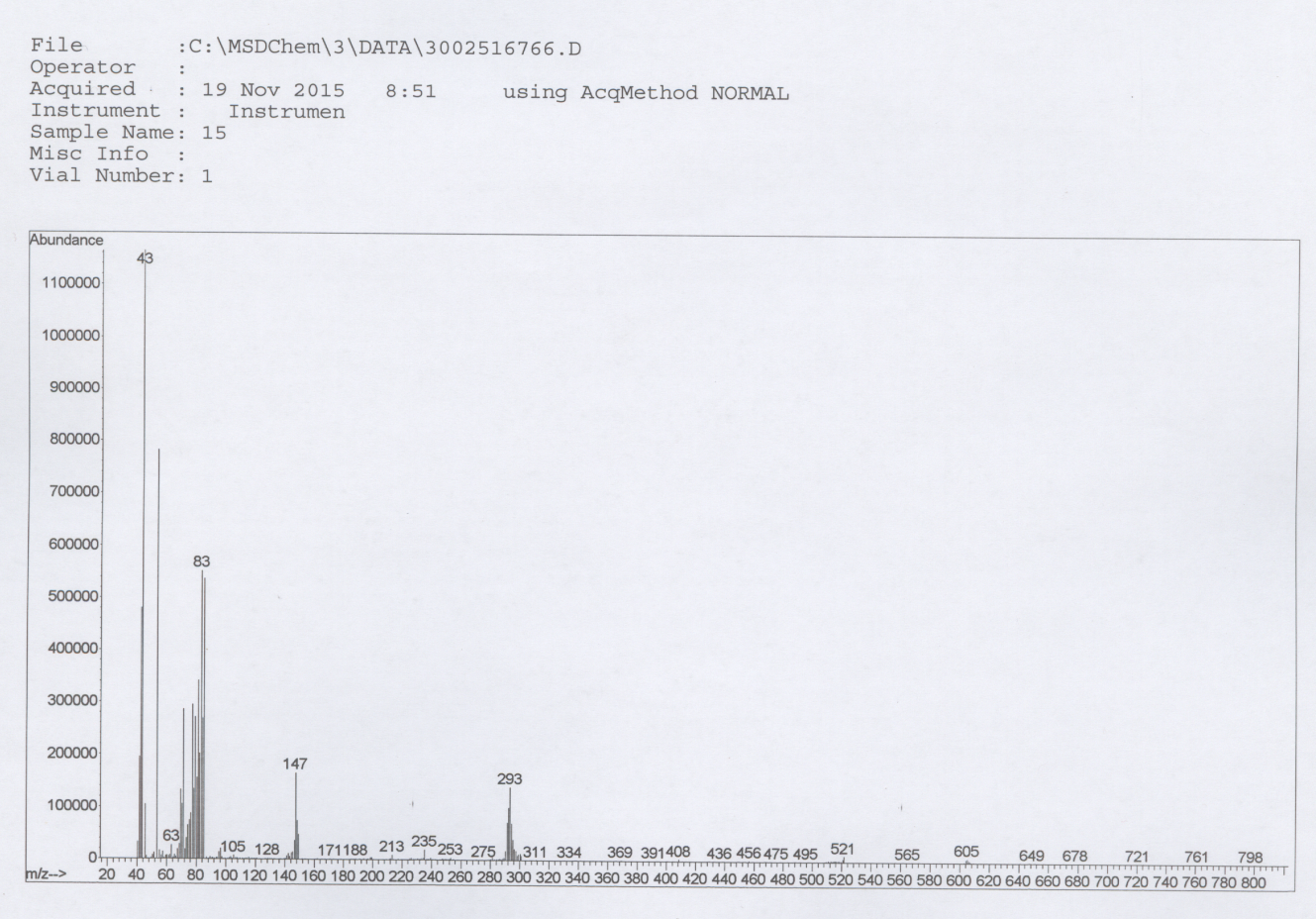


MS **2a**


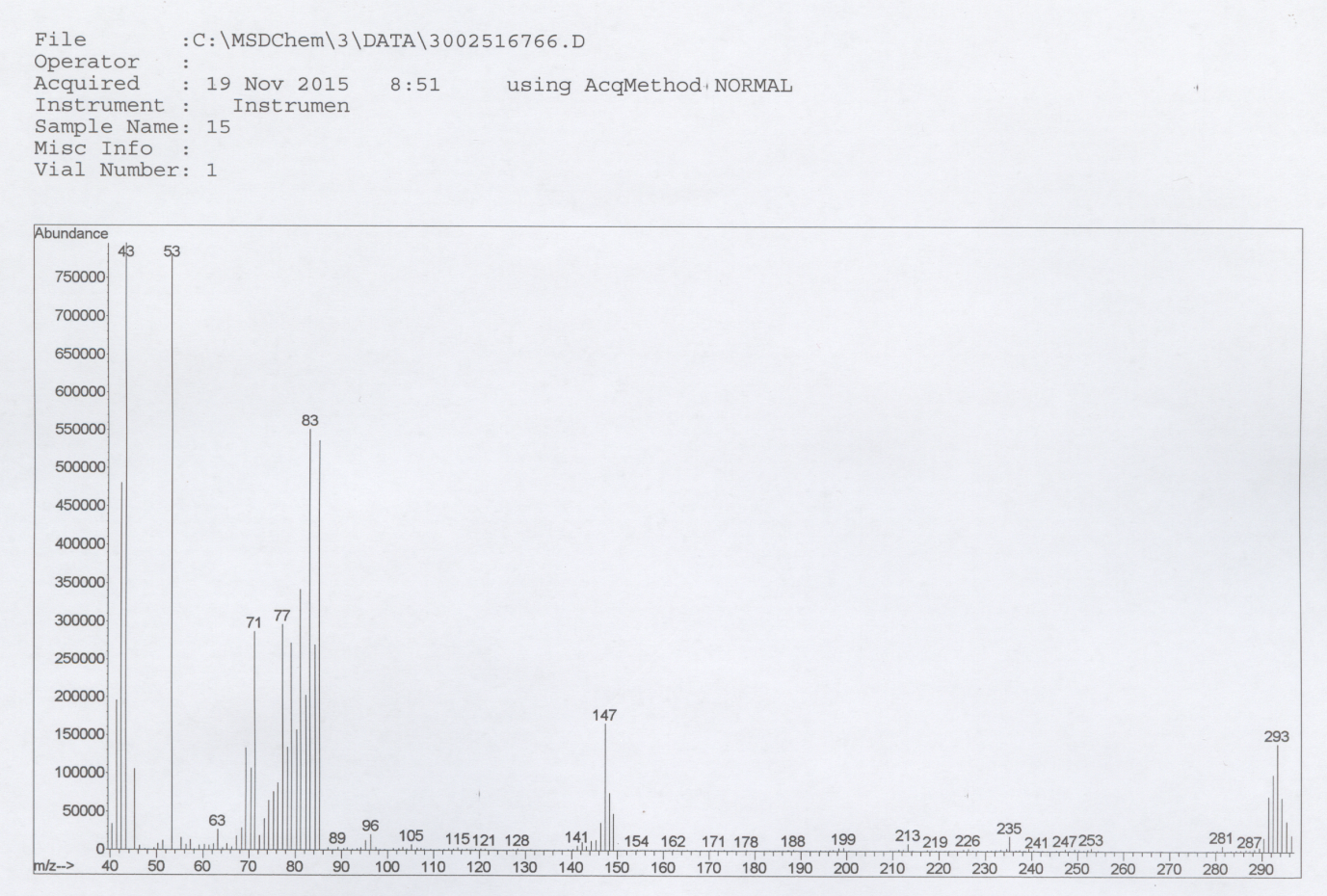


MS **2a**


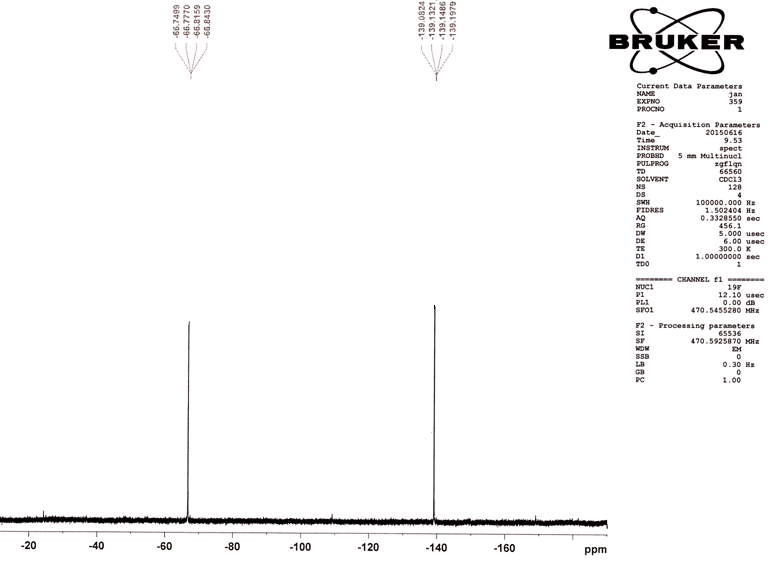


19F-NMR **3a**


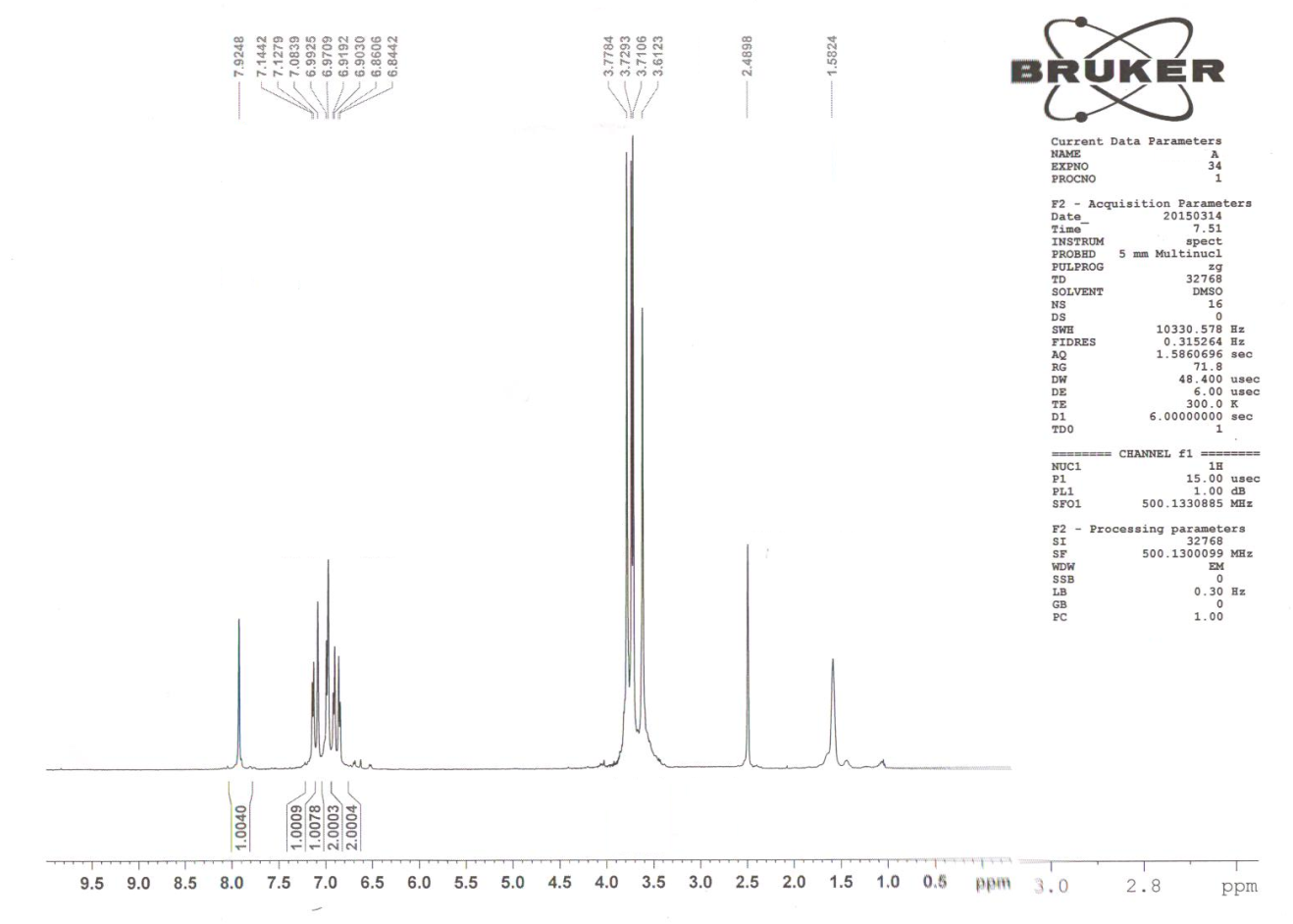


1H-NMR **3a**


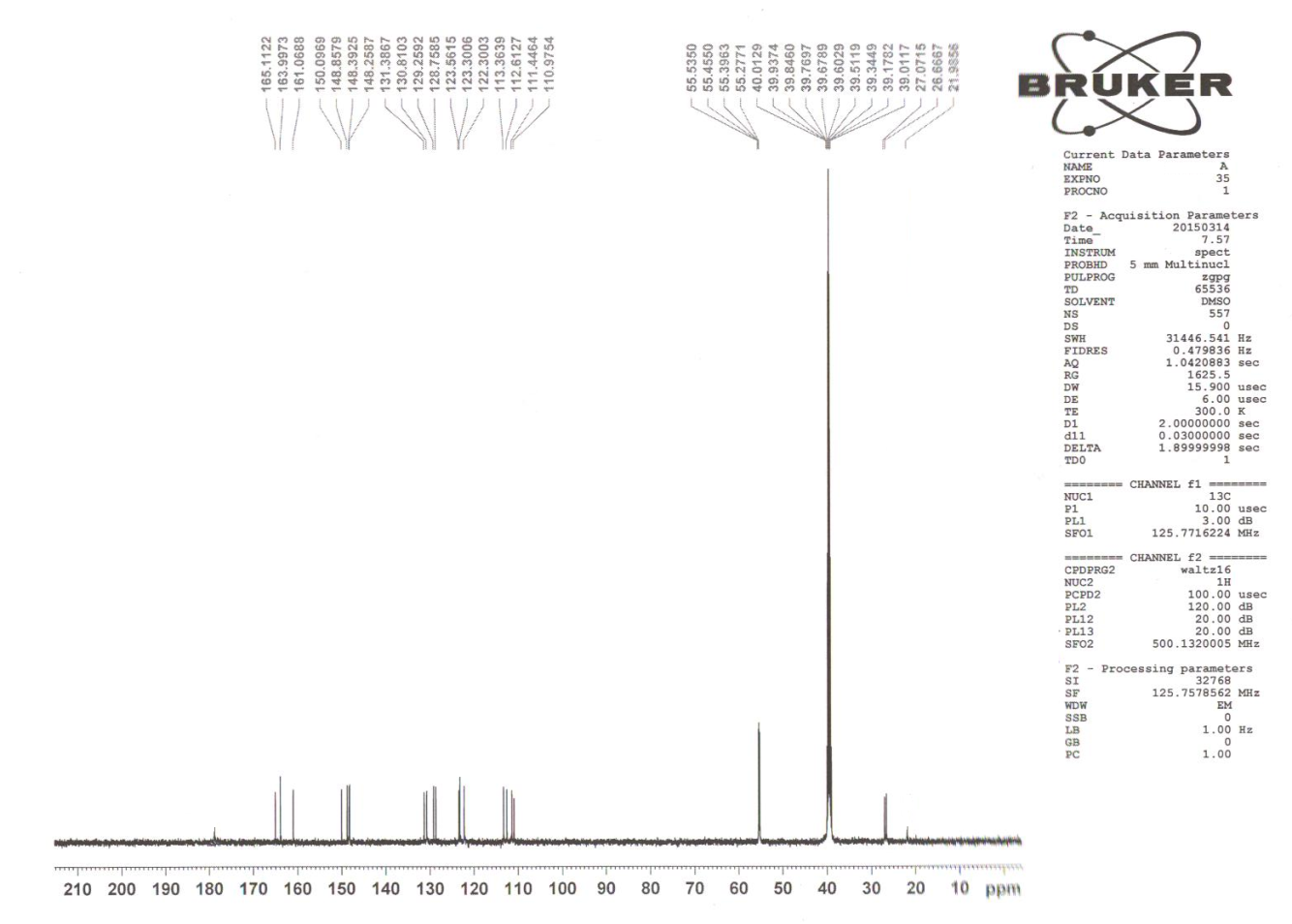


13C-NMR **3a**


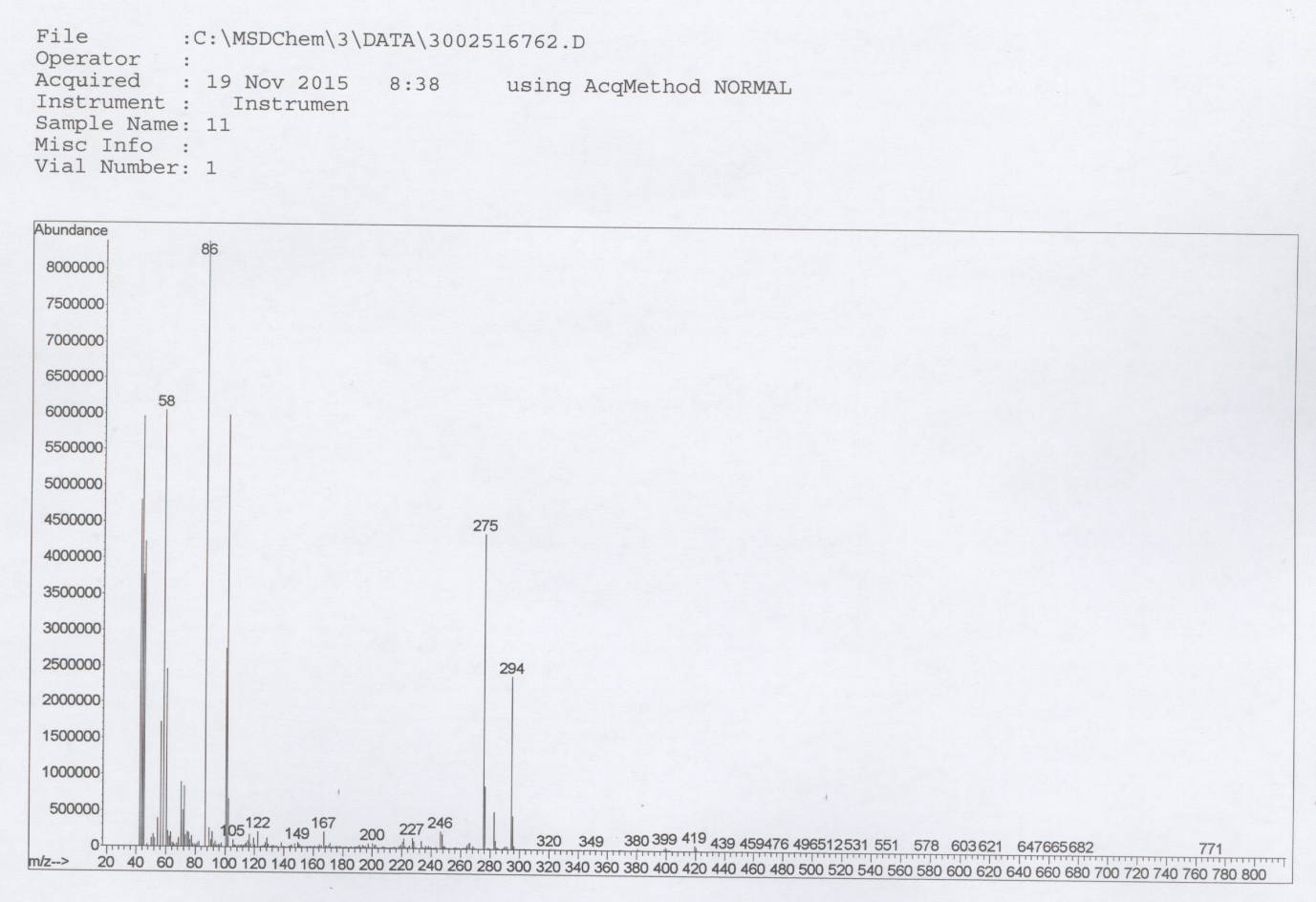


MS **3a**


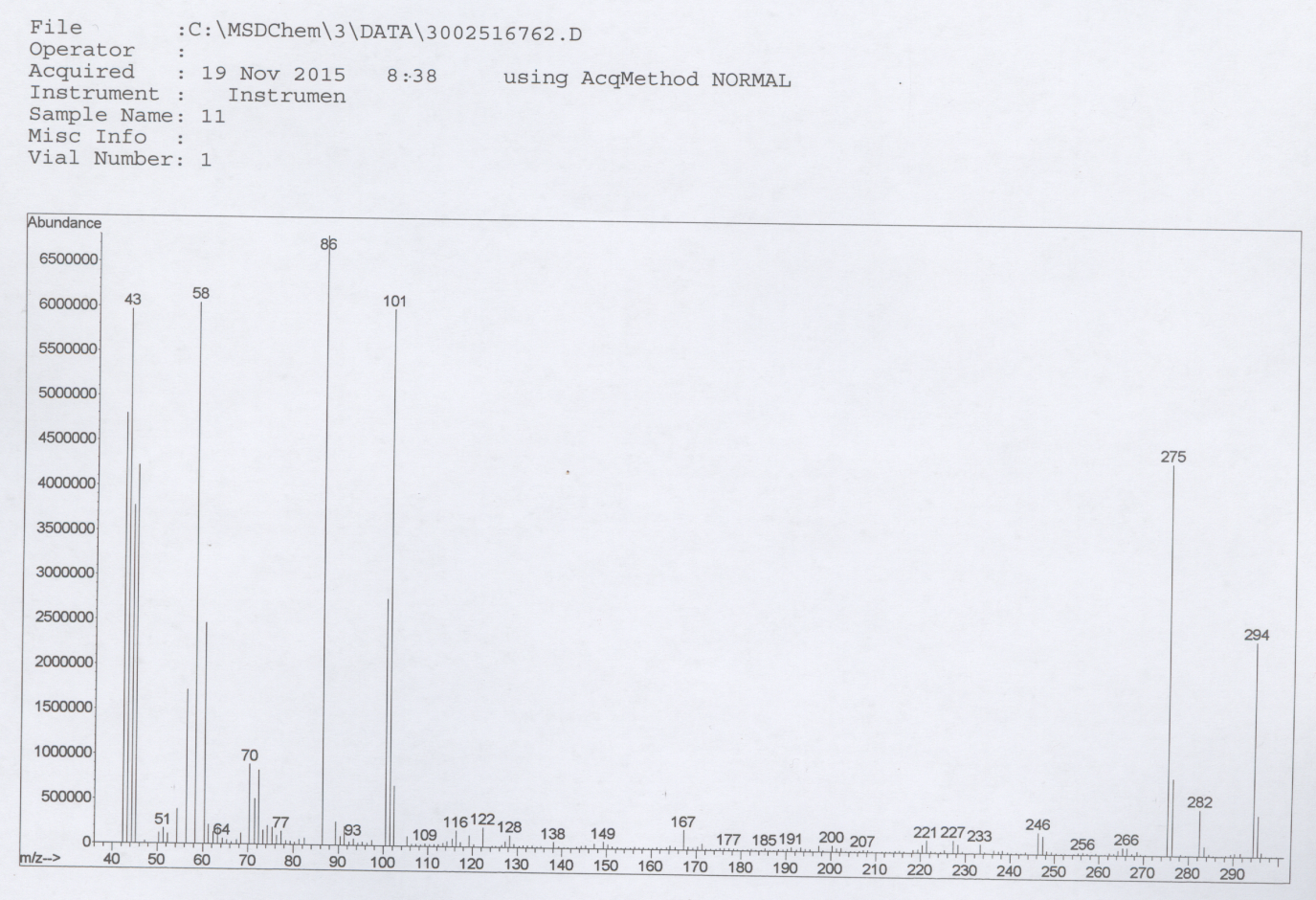


MS **3a**

Supplement: Supplementary file 1 — Additional file 1. 1H, 13C, 19F-NMR and MS spectra of the compounds. [file 40064_2016_3410_MOESM1_ESM.docx]
